# The satisfactory growth and development at 2 years of age of the INTERGROWTH-21^st^ Fetal Growth Standards cohort support its appropriateness for constructing international standards

**DOI:** 10.1016/j.ajog.2017.11.564

**Published:** 2018-02

**Authors:** José Villar, Leila Cheikh Ismail, Eleonora Staines Urias, Francesca Giuliani, Eric O. Ohuma, Cesar G. Victora, Aris T. Papageorghiou, Douglas G. Altman, Cutberto Garza, Fernando C. Barros, Fabien Puglia, Roseline Ochieng, Yasmin A. Jaffer, Julia A. Noble, Enrico Bertino, Manorama Purwar, Ruyan Pang, Ann Lambert, Cameron Chumlea, Alan Stein, Michelle Fernandes, Zulfiqar A. Bhutta, Stephen H. Kennedy, International Fetal and Newborn Growth Consortium for the 21st Century (INTERGROWTH-21st), M. Katz, M.K. Bhan, C. Garza, S. Zaidi, A. Langer, P.M. Rothwell, Sir D. Weatherall, Z.A. Bhutta, J. Villar, S. Kennedy, D.G. Altman, F.C. Barros, E. Bertino, F. Burton, M. Carvalho, L. Cheikh Ismail, W.C. Chumlea, M.G. Gravett, Y.A. Jaffer, A. Lambert, P. Lumbiganon, J.A. Noble, R.Y. Pang, A.T. Papageorghiou, M. Purwar, J. Rivera, C. Victora, M. Shorten, L. Hoch, H.E. Knight, E.O. Ohuma, C. Cosgrove, I. Blakey, E. Staines Urias, F. Roseman, N. Kunnawar, S.H. Gu, J.H. Wang, M.H. Wu, M. Domingues, P. Gilli, L. Juodvirsiene, N. Musee, H. Al-Jabri, S. Waller, D. Muninzwa, D. Yellappan, A. Carter, D. Reade, R. Miller, L. Salomon, A. Leston, A. Mitidieri, F. Al-Aamri, W. Paulsene, J. Sande, W.K.S. Al-Zadjali, C. Batiuk, S. Bornemeier, M. Dighe, P. Gaglioti, N. Jacinta, S. Jaiswal, K. Oas, M. Oberto, E. Olearo, M.G. Owende, J. Shah, S. Sohoni, T. Todros, M. Venkataraman, S. Vinayak, L. Wang, D. Wilson, Q.Q. Wu, Y. Zhang, P. Chamberlain, D. Danelon, I. Sarris, J. Dhami, C. Ioannou, C.L. Knight, R. Napolitano, S. Wanyonyi, C. Pace, V. Mkrtychyan, F. Al-Habsi, M. Alija, J.M. Jimenez-Bustos, J. Kizidio, F. Puglia, N. Kunnawar, H. Liu, S. Lloyd, D. Mota, R. Ochieng, C. Rossi, M. Sanchez Luna, Y.J. Shen, D.A. Rocco, I.O. Frederick, E. Albernaz, M. Batra, B.A. Bhat, E Bertino, P. Di Nicola, F. Giuliani, I. Rovelli, K. McCormick, V. Paul, V. Rajan, A. Wilkinson, A. Varalda, B. Eskenazi, L.A. Corra, H. Dolk, J. Golding, A. Matijasevich, T. de Wet, J.J. Zhang, A. Bradman, D. Finkton, O. Burnham, F. Farhi

**Affiliations:** a Nuffield Department of Obstetrics & Gynaecology and Oxford Maternal & Perinatal Health Institute, Green Templeton College, University of Oxford, Oxford, UK; b Centre for Statistics in Medicine, Nuffield Department of Orthopaedics, Rheumatology & Musculoskeletal Sciences, University of Oxford, Oxford, UK; c Department of Engineering Science, University of Oxford, Oxford, UK; d Department of Psychiatry, University of Oxford, Oxford, UK; e Clinical Nutrition and Dietetics Department, University of Sharjah, Sharjah, United Arab Emirates; f Azienda Ospedaliera OIRM Sant’Anna Citta della Salute e della Scienza di Torino, Torino, Italy; g Dipartimento di Scienze Pediatriche e dell’ Adolescenza, SCDU Neonatologia, Universita di Torino, Torino, Italy; h Programa de Pós-Graduação em Epidemiologia, Universidade Federal de Pelotasl, Pelotas, Brazil; i Programa de Pós-Graduação em Saúde e Comportamento, Universidade Católica de Pelotas, Pelotas, Brazil; j Division of Nutritional Sciences, Cornell University, Ithaca, NY; k Faculty of Health Sciences, Aga Khan University, Nairobi, Kenya; l Department of Family & Community Health, Ministry of Health, Muscat, Sultanate of Oman; m Nagpur INTERGROWTH-21^st^ Research Centre, Ketkar Hospital, Nagpur, India; n School of Public Health, Peking University, Beijing, China; o Lifespan Health Research Center, Boonshoft School of Medicine, Wright State University, Dayton, Ohio; p Center of Excellence in Women and Child Health, The Aga Khan University, Karachi, Pakistan; q Center for Global Child Health, Hospital for Sick Children, Toronto, Canada

**Keywords:** development, INTERGROWTH-21^st^ fetal growth standards, postnatal growth

## Abstract

**Background:**

The World Health Organization recommends that human growth should be monitored with the use of international standards. However, in obstetric practice, we continue to monitor fetal growth using numerous local charts or equations that are based on different populations for each body structure. Consistent with World Health Organization recommendations, the INTERGROWTH-21^st^ Project has produced the first set of international standards to date pregnancies; to monitor fetal growth, estimated fetal weight, Doppler measures, and brain structures; to measure uterine growth, maternal nutrition, newborn infant size, and body composition; and to assess the postnatal growth of preterm babies. All these standards are based on the same healthy pregnancy cohort. Recognizing the importance of demonstrating that, postnatally, this cohort still adhered to the World Health Organization prescriptive approach, we followed their growth and development to the key milestone of 2 years of age.

**Objective:**

The purpose of this study was to determine whether the babies in the INTERGROWTH-21^st^ Project maintained optimal growth and development in childhood.

**Study Design:**

In the Infant Follow-up Study of the INTERGROWTH-21^st^ Project, we evaluated postnatal growth, nutrition, morbidity, and motor development up to 2 years of age in the children who contributed data to the construction of the international fetal growth, newborn infant size and body composition at birth, and preterm postnatal growth standards. Clinical care, feeding practices, anthropometric measures, and assessment of morbidity were standardized across study sites and documented at 1 and 2 years of age. Weight, length, and head circumference age- and sex-specific z-scores and percentiles and motor development milestones were estimated with the use of the World Health Organization Child Growth Standards and World Health Organization milestone distributions, respectively. For the preterm infants, corrected age was used. Variance components analysis was used to estimate the percentage variability among individuals within a study site compared with that among study sites.

**Results:**

There were 3711 eligible singleton live births; 3042 children (82%) were evaluated at 2 years of age. There were no substantive differences between the included group and the lost-to-follow up group. Infant mortality rate was 3 per 1000; neonatal mortality rate was 1.6 per 1000. At the 2-year visit, the children included in the INTERGROWTH-21^st^ Fetal Growth Standards were at the 49th percentile for length, 50th percentile for head circumference, and 58th percentile for weight of the World Health Organization Child Growth Standards. Similar results were seen for the preterm subgroup that was included in the INTERGROWTH-21^st^ Preterm Postnatal Growth Standards. The cohort overlapped between the 3rd and 97th percentiles of the World Health Organization motor development milestones. We estimated that the variance among study sites explains only 5.5% of the total variability in the length of the children between birth and 2 years of age, although the variance among individuals within a study site explains 42.9% (ie, 8 times the amount explained by the variation among sites). An increase of 8.9 cm in adult height over mean parental height is estimated to occur in the cohort from low-middle income countries, provided that children continue to have adequate health, environmental, and nutritional conditions.

**Conclusion:**

The cohort enrolled in the INTERGROWTH-21^st^ standards remained healthy with adequate growth and motor development up to 2 years of age, which supports its appropriateness for the construction of international fetal and preterm postnatal growth standards.

Although human growth, from cell to whole body, is recognized as a universal biologic process, some entrenched views persist regarding fetal growth, in particular that it should be compared with a site-specific rather than prescriptive population. This view is not held by the World Health Organization (WHO) or by the Centers for Disease Control & Prevention,^1^, ^2^ which recommend using international neonatal standards. Likewise, such standards have now been adopted to estimate the burden and consequences of babies being born small for gestational age in low- and middle-income countries.^3^

We have summarized the key statistical, physiologic, ethnic, and genetic evidence relating to this issue.^4^, ^5^ Practically, the debate focuses on whether it is correct to monitor fetal growth using 1 of the many site-specific charts available. Typically, such charts are based on different populations for each fetal body structure and have been developed at hospital level.^4^ These multiple, site-specific charts are references, not international standards that are used commonly in most other areas of biology and medicine.

This neglected aspect of obstetric practice means that clinical decisions are made based on reference charts that were derived from a wide range of different study populations. For example, a woman may have an early gestational age assessment with the use of a fetal crown-rump length chart based on a study of 80 women from Glasgow, Scotland,^6^, ^7^ followed by a clinical assessment with the use of a fundal height chart based on 313 women from Cardiff, Wales.^8^ Fetal biometry values may then be compared with 1 of many local charts,^9^ and, during the same ultrasound scan, estimated fetal weight may be determined from an equation based on 109 fetuses studied in Texas during the 1980s,^10^, ^11^ complemented by a recent chart from other US populations.^12^

If the woman requires further assessment, the umbilical Doppler measures are judged with the use of yet another reference population.^13^ At birth, the anthropometric measures of the newborn infant could be evaluated with the use of a multiplicity of reference charts, all of which are totally unrelated to the fetal growth charts that were being used just a few weeks earlier.

The INTERGROWTH-21^st^ Project aimed to resolve these issues by conducting studies of human growth and development that involved pregnant women who were enrolled at <14 weeks gestation specifically to monitor their fetuses, newborn infants, and children prospectively up to 2 years of age to generate a single set of international standards to make judgements on the growth of all humans.^14^ The studies were based conceptually on the WHO prescriptive approach to constructing human growth standards.^15^ The study populations across geographically delimited areas were selected because they had the recommended health, nutrition, and socioeconomic status that was required to construct international standards.^15^

Hence, the INTERGROWTH-21^st^ Standards (from maternal weight gain, to pregnancy dating, fetal growth and estimated fetal weight, to brain structures, amniotic fluid volume, umbilical artery Doppler measures, and newborn body composition) are prescriptive because they are based on a cohort of “healthy” pregnancies and babies from the same geographically selected populations in which most of the health and nutritional needs of mothers were met and adequate antenatal care provided.

Nevertheless, the question always remains with studies that are focused on fetal growth as to how “healthy” were these children after birth and during childhood (ie, are they truly healthy?). We took this question seriously very early in the planning of the project and added a clinical and developmental follow-up evaluation^16^, ^17^, ^18^ beyond the customary early neonatal period as a further criterion to support the assertion that INTERGROWTH-21^st^ babies represent true standard populations.^19^ The key milestone of 2 years of age was identified as a realistic and biologically relevant time point.^20^

Hence, we first compared the INTERGROWTH-21^st^ Standards^4^, ^21^, ^22^ with the WHO Child Growth Standards.^23^ We demonstrated that, during the early neonatal period, the participants who were selected were appropriate and met the WHO prescriptive criteria for optimal growth.^15^ We then extended, for the first time in this literature, the prescriptive evaluation by designing the Infant Follow-up Study of the INTERGROWTH-21^st^ Project.

This study aimed to evaluate the growth, nutrition, morbidity, and motor development at 2 years of age of the infants who were included in the international fetal and preterm growth standards to reinforce their prescriptive nature against which fetuses and preterm infants worldwide can now be compared.

## Materials and Methods

INTERGROWTH-21^st^ was a multicenter, population-based project that was conducted between 2009 and 2016 in 8 locations: Pelotas, Brazil; Turin, Italy; Muscat, Oman; Oxford, UK; Seattle, WA; Shunyi County, Beijing, China; the central area of Nagpur, India, and the Parklands suburb of Nairobi, Kenya.^14^, ^24^

The primary aim of the project was to study growth, health, nutrition, and neurodevelopment from <14 weeks gestation to 2 years of age.^14^ In the Fetal Growth Longitudinal Study of the INTERGROWTH-21^st^ Project,^21^ we recruited women from these 8 populations who initiated antenatal care at <14 weeks gestation and who met the entry criteria of optimal health, nutrition, education, and socioeconomic status.^14^

Gestational age was estimated based on the date of the last menstrual period and corroborated by ultrasound measurement of crown-rump length at 9^+0^ to 13^+6^ weeks gestation with the use of a standard protocol. All fetuses in the Fetal Growth Longitudinal Study were eligible to contribute data to the construction of the international fetal growth standards; all infants who were born at <37 weeks gestation in the Fetal Growth Longitudinal Study were eligible to contribute data to the construction of the international Postnatal Growth Standards for Preterm Infants. At each postnatal visit, a record of any illnesses in the preceding months was noted in addition to anthropometric measurements and a developmental assessment.

Weight, length, and head circumference were obtained within 12 hours (and no >24 hours) of birth on the postnatal wards and at follow-up visits that were scheduled at 1 and 2 years of age (±1 month). Measurements were taken exclusively by the same teams who were trained and standardized at regular intervals for the INTERGROWTH-21^st^ Project.^25^

All study sites used the same methods and equipment: electronic scales (Seca, Hangzhou, China) for weight (sensitivity of 10 g to 20 Kg); a specially designed Harpenden infantometer (Chasmors Ltd, London, UK) for recumbent length, and a metallic nonextendable tape (Chasmors Ltd) for head circumference.^26^, ^27^ Measurement procedures were standardized according to WHO recommendations.^28^ During the central standardization sessions for anthropometrists, the intra- and interobserver error of measurement values for recumbent length ranged from 0.3–0.6 cm and for head circumference from 0.2–0.5 cm.^25^

Measurements were taken twice, independently, by 2 of the study anthropometrists. If the difference between the 2 measures exceeded for weight 50 g for newborn infants and ≤100 g at 1 and 2 years of age (length, 7 mm; head circumference, 5 mm), then both observers independently repeated that measurement a second time and, if necessary, a third time.^25^, ^27^

When the Infant Follow-up Study started, some enrolled children had passed their second birthday already. The families of these children were invited to a follow-up visit with the maximum age at assessment for the child being 27 months. Similarly, those children who already had passed their first birthday, but were <2 years old, were invited initially for the first visit up to the age of 18 months. In total, only 14% of 1- and 2-year visits occurred outside the protocol-designated age range for assessment.

Detailed information was obtained from the mother about the infant’s health, severe morbidities, length of breastfeeding, timing of the introduction of food, feeding practices, and food intake with the use of standardized forms that were produced especially for the project (www.intergrowth21.org).

WHO protocols were followed to assess motor development milestones.^29^ We focused on 4 WHO milestones that are less likely to be affected by recall bias: sitting without support, hands and knees crawling, standing alone, and walking alone. Data were collected by trained staff using a form with pictures of the relevant child positions and corresponding definitions. Parents were asked to report the age in months and weeks when they first observed or “never observed” the milestones (http://www.intergrowth21.org.uk).

We collected the same information from parents at 1 and 2 years of age to evaluate the consistency of the reported dates. There were 7965 pairs of values recorded at year 1 and the year 2 interviews, of which 92.6% were identical at both visits. Among the 588 discrepant values, the median difference ranged between –1 week (interquartile range, −4.3–4.3) for hands and knees crawling to –0.2 weeks (interquartile range, –6.3–2.3) for standing alone. In these cases, after investigation, the values that were obtained at the 1-year visit were used.

Across all study sites, standardized clinical care and feeding practices were implemented based on protocols that were developed by the INTERGROWTH-21^st^ Neonatal Group (http://www.intergrowth21.org.uk).^30^, ^31^, ^32^ Exclusive breastfeeding up to 6 months was promoted for all babies, with supplementation for preterm infants as recommended.^30^, ^33^, ^34^

Age- and sex-specific z-scores and percentiles were estimated for each child at 2 years of age comparing their weight, length, and head circumference to the WHO Child Growth Standards.^35^ Corrected age was used for the preterm subgroup.^36^ Four values (3 for weight and 1 for head circumference) were above or below 5 standard deviations (SD) of the mean of the study population and were excluded.

Variance components analysis was performed to calculate the percentage of variance in infant length at birth, 1, and 2 years because of between- and within-site variance. A multilevel mixed effects model was fitted with random intercepts for the study site and individual levels (with individuals nested within sites). The model, which was fitted with unstructured covariance structure, was adjusted by age (after fractional polynomial transformation) and sex. Both age and sex were treated as fixed effects.

We analyzed 2026 mother-father-infant trios to compare the “mean parental height” with a predicted adult height for each infant, defined as twice their length at 2 years of age.^37^

For infants reported to have achieved the milestones, the proportions within the WHO motor development windows of achievement^35^ were estimated, and z-scores were calculated by subtraction of the median age of achievement reported in the WHO motor development study from the median age of achievement in our cohort, and division by the SD in the WHO motor development study. Corrected age was used for the preterm subgroup.

The proportion of infants who received breast milk and vitamin and mineral supplements and those who followed a special diet were estimated at 1 and 2 years of age.^38^, ^39^

We used Stata software (version 12; StataCorp, College Station, TX). Data were entered locally into the specially developed online data management system (http://medscinet.com).^40^

The INTERGROWTH-21^st^ Project was approved by the Oxfordshire Research Ethics Committee “C” (reference: 08/H0606/139), the research ethics committees of the individual institutions, and the regional health authorities where the project was implemented. Participants provided written consent to be involved in the study.

## Results

### Population characteristics

**Figure 1:**
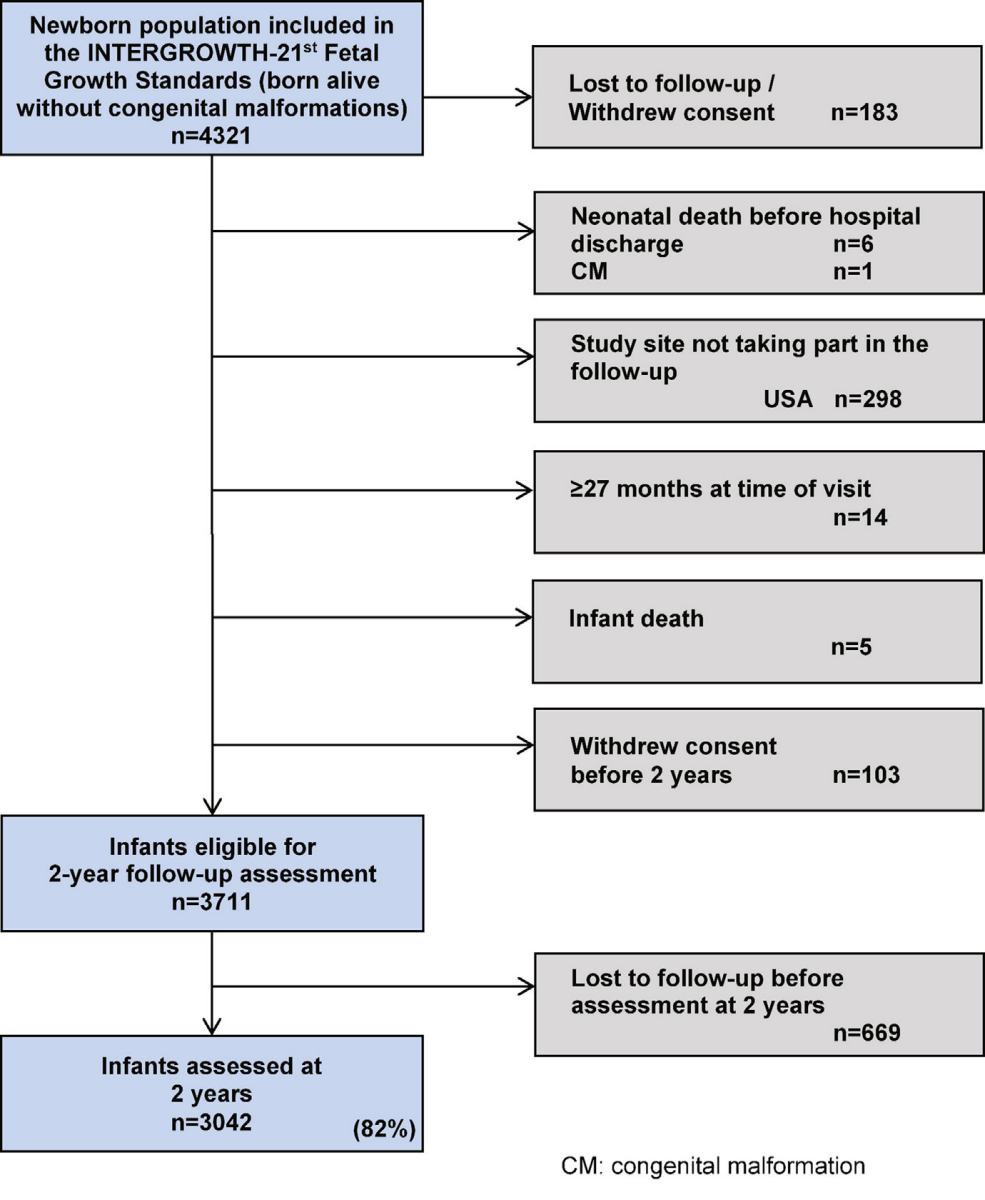
Study flow of the INTERGROWTH-21^st^ Infant Follow-up Study The chart indicates the cohort that contributed data to the construction of the INTERGROWTH-21^st^ Fetal Growth Standards.^21^ *CM*, congenital malformation; *USA*, United States of America. *Villar et al. Validation of the INTERGROWTH-21^st^ fetal growth standards. Am J Obstet Gynecol 2018*.

There were 4321 singleton newborn infants who were alive at birth without congenital malformations whose mothers were recruited at <14 weeks gestation and included in the cohort of the international INTERGROWTH-21^st^ fetal growth standards.^21^ Among these, 183 infants were lost to follow up or withdrew consent during pregnancy; 298 infants were ineligible for the Infant Follow-up Study because the study site in Seattle, WA, could not participate. There were 6 neonatal deaths before hospital discharge (neonatal mortality rate, 1.6/1000 live births), 1 congenital malformation that was detected after birth, and 5 infant deaths, which represented a total infant mortality rate of 3 per 1000 live births. In addition, 103 mothers withdrew consent early in the study. Finally, 14 infants were >27 months old at the time the follow-up started; they therefore were not invited to participate. Hence, 3711 newborn infants were eligible for the Infant Follow-up Study, of these, 669 infants were lost to follow up. Thus, the total cohort that was studied comprised 3042 infants (Figure 1) who represented 82% of those eligible (86% for the preterm subgroup, 143/166; Supplementary Figure).

The means (±SD) of the age at which measures were obtained were 24.4±1.2 and 23.2±0.7 months for the total cohort and the preterm subgroup, respectively; 86% of the 2-year measures were obtained from 23–25 months for the total cohort and 93% were obtained for the preterm subgroup.

The neonatal characteristics of the infants divided into those that completed the 2-year follow-up evaluations (n=3042) and those lost to follow up (n=669) are presented in Table 1. Both groups were similar in terms of anthropometric measures at birth and neonatal morbidity. A similar comparison within the preterm subgroup is presented in Table 2.

**Table 1:** Neonatal characteristics of children who were included in the INTERGROWTH-21^st^ Fetal Growth Standards^21^ who were evaluated at 2 years of age compared with children who were lost to follow-up

Characteristic	Evaluated at 2 years of age (n=3042)	Not evaluated at 2 years of age^a^ (n=669)
Gestational age at delivery, wk^b^	39.4±1.4	39.4±1.4
Birthweight, kg^b^	3.2±0.5	3.2±0.5
Birth length, cm^b^	49.1±2.0	49.2±2.0
Head circumference, cm^b^	33.7±1.4	33.9±1.3
Apgar at 5 min^b^	9.6±0.6	9.7±0.6
Age at hospital discharge, d^c^	3 (2–4)	2 (1–4)
Early preterm, <34 wk gestation, n (%)	18 (0.6)	3 (0.4)
Boys, n (%)	1516 (49.8)	324 (48.4)
Neonatal intensive care unit stay >1 d but <3 d, n (%)	160 (5.3)	35 (5.2)
Hyperbilirubinemia, n (%)	137 (4.5)	37 (5.5)
Respiratory distress syndrome, n (%)	51 (1.7)	15 (2.2)
Transient tachypnea of the newborn infant, n (%)	65 (2.1)	15 (2.2)
Exclusive breastfeeding at discharge, n (%)	2698 (88.8)	591 (88.5)

*Villar et al. Validation of the INTERGROWTH-21^st^ fetal growth standards. Am J Obstet Gynecol 2018*.

a Children lost to follow-up before evaluation at 2 years of age

b Data are means±standard deviation

c Data are given as median (interquartile range).

**Table 2:** Neonatal characteristics of children who were included in the INTERGROWTH-21^st^ Preterm Postnatal Growth Standards^22^ who were evaluated at 2 years of age compared with children lost to follow-up

Characteristic	Evaluated at 2 years of age (n=143)	Not evaluated at 2 years of age^a^ (n=24)
Gestational age at delivery, wk^b^	35.5±1.6	35.7±1.4
Birthweight, kg^b^	2.5±0.5	2.4±0.5
Birth length, cm^b^	45.7±2.7	45.6±2.3
Head circumference, cm^b^	31.8±1.7	31.8±1.5
Apgar at 5 min^b^	9.2±0.9	9.2±1.2
Age at hospital discharge, d^c^	4 (2–9)	4 (2–7)
Early preterm, <34 weeks gestation, n (%)	19 (13.3)	3 (12.5)
Boys, n (%)	73 (51.0)	8 (33.3)
Neonatal intensive care unit stay >1 but <3 d, n (%)	59 (41.3)	11 (45.8)
Hyperbilirubinemia, n (%)	29 (20.3)	3 (12.5)
Respiratory distress syndrome, n (%)	20 (14.0)	6 (25.0)
Transient tachypnea of the newborn infant, n (%)	23 (16.1)	1 (4.2)
Exclusive breastfeeding at discharge, n (%)	106 (74.1)	19 (79.2)

*Villar et al. Validation of the INTERGROWTH-21^st^ fetal growth standards. Am J Obstet Gynecol 2018*.

a Children lost to follow-up before evaluation at 2 years of age

b Data are given as mean±standard deviation

c Data are given as median (interquartile range).

### Feeding practices

At hospital discharge, 89% of the total cohort and 74% of the preterm subgroup were exclusively breast-milk fed. Similar patterns were seen among the children who were lost to follow-up at 2 years of age. Exclusive breastfeeding was stopped at a median of 5 months (interquartile range, 3–6 months); this was similar in the preterm subgroup. Breastfeeding stopped entirely at a median of 12 months (interquartile range, 6–18 months) for the total cohort and 11 months (interquartile range, 5–18 months) for the preterm subgroup.

In the total cohort, the proportion of children who still were receiving breast milk fell from 59% at 1 year to 11% at 2 years, by which time 34% of the children were formula fed. All children received dairy products of some type (including human milk) at both ages. Food supplements had been given routinely to 33% of children by 1 year and 21% by 2 years. At 1 year of age, 51% of the infants in the preterm subgroup were still receiving breast milk; the figure fell to 8% at 2 years, by which time 34% of children were receiving formula. Food supplements that included vitamins and minerals were given to 36% of the infants in the first year and 28% of the infants by the second year in the preterm cohort. Complementary feeding practices were considered appropriate in terms of diversity, the timing of introduction, and the food variety across sites (Supplementary Tables 1 and 2).^34^

### Postnatal morbidity

The overall morbidity rate in the total cohort was low (Table 3); only 9% of infants were hospitalized (median length of stay, 3 days) in the second year of life. The most frequently reported or diagnosed conditions were acute respiratory infections, diarrhea, and/or gastrointestinal problems with few repeated episodes, skin problems, and febrile episodes. Antibiotics were prescribed on >3 occasions in 10.9% and 15.8% of children in the first and second years, respectively, which corresponds closely to the rate of reported fever episodes (Table 3). Similar patterns were seen in the preterm subgroup (Table 4). Most of the infants were fully vaccinated in accordance with recommended policies.

**Table 3:** Morbidity in the previous year of children who were included in the INTERGROWTH-21^st^ Fetal Growth Standards^21^ at 1 and 2 years of age

Medical condition	1 Year of age (n=2834), n (%)	2 Years of age (n=3042), n (%)
Hospitalized at least once	344 (12.1)	272 (8.9)
Total no. of days hospitalized	3 (1–5)^a^	3 (1–5)^a^
Any prescription made by a healthcare professional	1783 (62.9)	1911 (62.9)
Antibiotics (≥3 regimens)	308 (10.9)	481 (15.8)
Iron/folic acid/vitamin B12/other vitamins	815 (28.8)	430 (14.1)
Up-to-date with local vaccination policies	2607 (92.0)	2903 (95.4)
Otitis media/pneumonia/bronchiolitis	228 (8.0)	293 (9.6)
Parasitosis/diarrhea/vomiting	148 (5.2)	139 (4.6)
Seizures/cerebral palsy/neurologic disorders	9 (0.3)	9 (0.3)
Exanthema/skin disease	456 (16.1)	399 (13.1)
UTI/pyelonephritis	4 (0.1)	10 (0.3)
Fever ≥3 d (≥3 episodes)	293 (10.3)	309 (10.2)
Malaria	13 (0.5)	12 (0.4)
Meningitis	5 (0.2)	0 (0.0)
Other infections that required antibiotics	69 (2.4)	79 (2.6)
Hearing problems	4 (0.1)	3 (0.1)
Asthma	24 (0.8)	42 (1.4)
Cardiovascular problems	9 (0.3)	7 (0.2)
Blindness	6 (0.2)	4 (0.1)
Gastroesophageal reflux	88 (3.1)	9 (0.3)
Any hemolytic condition	14 (0.5)	22 (0.7)
Any malignancy	3 (0.1)	6 (0.2)
Cow’s milk protein allergy	NA	21 (0.7)
Food allergies	NA	52 (1.7)
Injury trauma	43 (1.5)	130 (4.3)
Any condition that required surgery	31 (1.1)	34 (1.1)

*NA*, not applicable (data were not collected at the 1-year follow-up visit); *UTI*, urinary tract infection.

*Villar et al. Validation of the INTERGROWTH-21^st^ fetal growth standards. Am J Obstet Gynecol 2018*.

a Data are given as median (interquartile range).

**Table 4:** Morbidity of children who were included in the INTERGROWTH-21^st^ Preterm Postnatal Growth Standards^22^ at 1 and 2 years of age

Medical condition	1 Year of age (n=154), n (%)	2 Years of age (n=143), n (%)
Hospitalized at least once	34 (22.1)	7 (4.9)
Total number of days hospitalized	5 (3–8)^a^	7 (3–9)^a^
Any prescription made by a healthcare professional	98 (63.6)	72 (50.3)
Antibiotics (≥3 regimens)	31 (20.1)	12 (8.4)
Iron/folic acid/vitamin B12/other vitamins	56 (36.4)	23 (16.1)
Up-to-date with local vaccination policies	139 (90.3)	136 (95.1)
Otitis media/pneumonia/bronchiolitis	13 (8.4)	7 (4.9)
Parasitosis/diarrhea/vomiting	11 (7.1)	10 (7.0)
Seizures/cerebral palsy/neurologic disorders	1 (0.6)	0
Exanthema/skin disease	27 (17.5)	21 (14.7)
UTI/pyelonephritis	0	0
Fever ≥3 d (≥3 episodes)	11 (7.1)	5 (3.5)
Malaria	0	1 (0.7)
Meningitis	0	0
Other infections that required antibiotics	2 (1.3)	4 (2.8)
Hearing problems	0 (0.0)	0
Asthma	2 (1.3)	1 (0.7)
Cardiovascular problems	0	1 (0.7)
Blindness	0	0
Gastroesophageal reflux	6 (3.9)	0
Any hemolytic condition	2 (1.3)	2 (1.4)
Any malignancy	1 (0.6)	0
Cow’s milk protein allergy	NA	3 (2.1)
Food allergies	1 (0.6)	3 (2.1)
Injury trauma	1 (0.6)	4 (2.8)
Any condition that required surgery	2 (1.3)	4 (2.8)

*Villar et al. Validation of the INTERGROWTH-21^st^ fetal growth standards. Am J Obstet Gynecol 2018*.

*NA*, not applicable (data were not collected at the 1-year follow-up visit); *UTI*, urinary tract infection.

a Data are given as median (interquartile range).

### Growth and development from birth to 2 years of age

At 1 year of age, a comparison of the total cohort with the age- and sex-specific WHO Child Growth Standards showed that length and head circumference had a mean ± SD z-score of 0.0±1.1 for both measures and that the medians were at the 49th and 48th percentiles of the WHO Child Growth Standards, respectively; for weight, the mean z-score was 0.2±1.1 and median at the 58th percentile.

**Figure 2:**
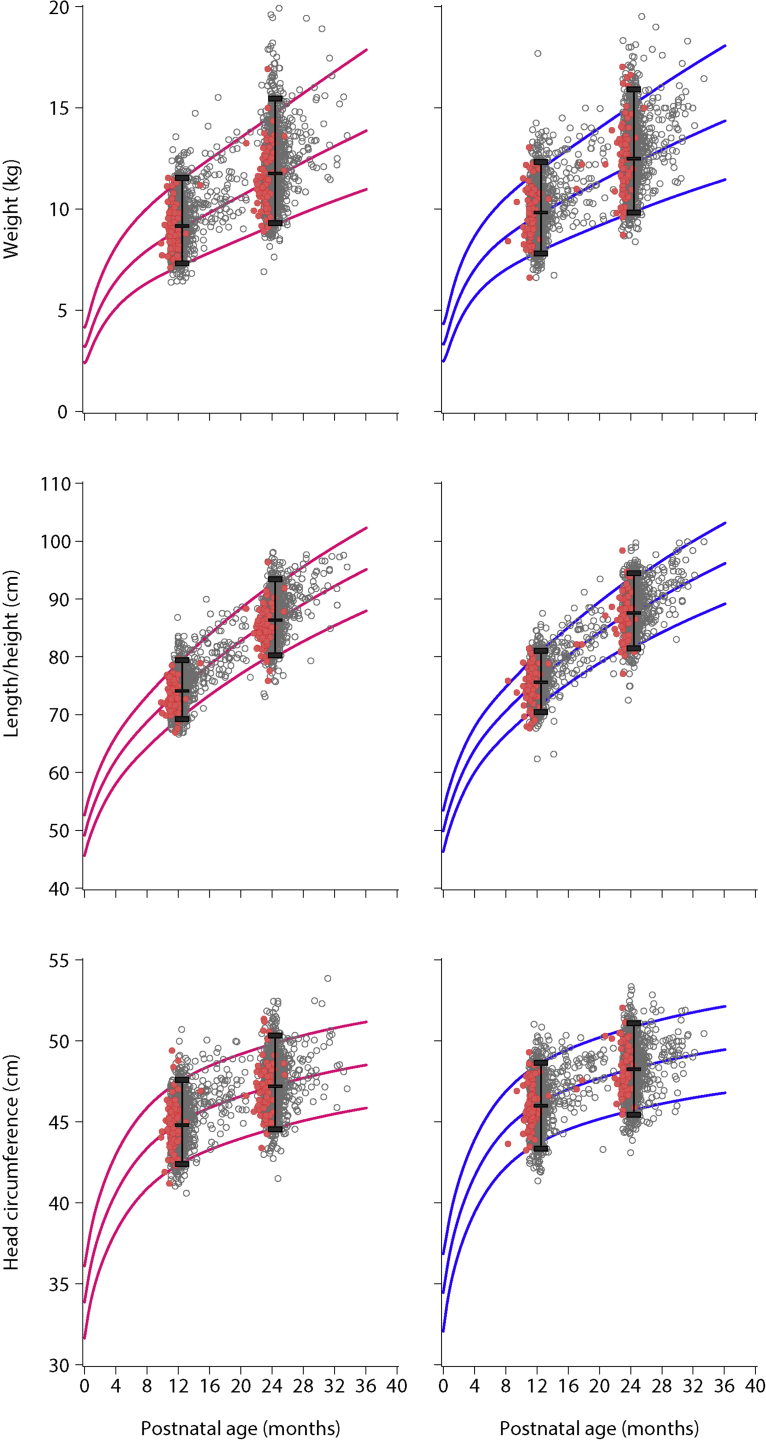
Anthropometric measures at 1 and 2 years of age of the children included in the INTERGROWTH-21^st^ Fetal Growth Standards Data are for children who were included in the INTERGROWTH-21^st^ Fetal Growth Standards^21^ (*grey circles*) and children who were included in the Preterm Postnatal Growth Standards^22^ (*red circles*). Values are superimposed onto the 3rd, 50th, and 97th percentiles of the World Health Organization Child Growth Standards^23^ (girls [*pink lines*] and boys [*blue lines*]). For children born preterm, corrected postnatal age was used. *Villar et al. Validation of the INTERGROWTH-21^st^ fetal growth standards. Am J Obstet Gynecol 2018*.

At the 2-year visit, the growth of the children who were included in the INTERGROWTH-21^st^ Fetal Growth Standards plotted almost perfectly onto the WHO Child Growth Standards (ie, 93% for length, 91% for weight [with the expected larger variability], and 92% for head circumference, respectively. Our cohort’s values were within the 3rd and 97th cut-off points of the WHO Child Growth Standards (Table 5; Figure 2). For length and head circumference, the mean ± SD z-score was 0.0±1.1 for both measures, and the medians were at the 49th and 50th percentiles of the WHO Child Growth Standards, respectively. For weight, the mean ± SD z-score was 0.2±1.1, and median was at the 58th percentile. Figure 2 also shows the 3rd, 50th and 97th percentiles of the distributions of our data (the same percentiles of the WHO Child Growth Standards are included in Figure 2 at years 1 and 2). As shown, the percentiles from our population are almost identical to those of the WHO standards.

**Table 5:** Anthropometric measures at 2 years of age of children who were included in the INTERGROWTH-21^st^ Fetal Growth Standards^21^ compared with the World Health Organization Child Growth Standards^a^

Variable	N	INTERGROWTH-21^st^		World Health Organization Child Growth Standards	
		Mean±standard deviation^b^	Median (interquartile range)	Mean z-score±standard deviation	Median percentile
Weight, kg	3025	12.3±1.7	12.2 (11.1–13.3)	0.2±1.1	58
Length, cm	3010	87.4±3.6	87.3 (85.0–89.7)	0.0±1.1	49
Head circumference, cm	3003	47.8±1.6	47.8 (46.7–48.8)	0.0±1.1	50

*Villar et al. Validation of the INTERGROWTH-21^st^ fetal growth standards. Am J Obstet Gynecol 2018*.

a Age and gender-specific z-scores and percentiles compared with the World Health Organization Child Growth Standards^23^

b Mean values were estimated from raw data.

At 1 year of age, a comparison of the preterm cohort only with the age- and sex-specific WHO Child Growth Standards at postnatal corrected age, length, and head circumference had a mean z-score of 0.1 for both measures; the medians were at the 52nd percentiles of the WHO Child Growth Standards; for weight, the mean ± SD z-score was 0.2±1.1, and the median was at the 57th percentile.

At 2 years of age, the growth of the children who were included in the INTERGROWTH-21^st^ Preterm Postnatal Growth Standards also plotted similarly onto the WHO Child Growth Standards (Table 6; Figure 2). For length and head circumference, the mean ± SD z-scores were -0.1±1.2 and 0.0±1.1, respectively, and the median was at the 47th percentile for head circumference of the WHO Child Growth Standards for both measures. For weight, the mean ± SD z-score was 0.2±1.1, and the median was at the 53rd percentile.

**Table 6:** Anthropometric measures at 2 years of age of children who were included in the INTERGROWTH-21^st^ Preterm Postnatal Growth Standards^22^ compared with the World Health Organization Child Growth Standards^a^

Variable	N	INTERGROWTH-21^st^		Comparison with World Health Organization Child Growth Standards	
		Mean±standard deviation^b^	Median (interquartile range)	Mean z-score±standard deviation	Median percentile
Weight, kg	142	12.0±1.7	11.7 (10.8–13.2)	0.2±1.1	53
Length, cm	141	86.2±3.7	86.2 (83.8–88.3)	–0.1±1.2	47
Head circumference, cm	140	47.7±1.6	47.6 (46.7–48.6)	0.0±1.1	47

*Villar et al. Validation of the INTERGROWTH-21^st^ fetal growth standards. Am J Obstet Gynecol 2018*.

a Corrected age was used to obtain age and gender-specific z-scores and percentiles comparing to the World Health Organization Child Growth Standards^23^

b Mean values were estimated from raw data.

**Figure 3:**
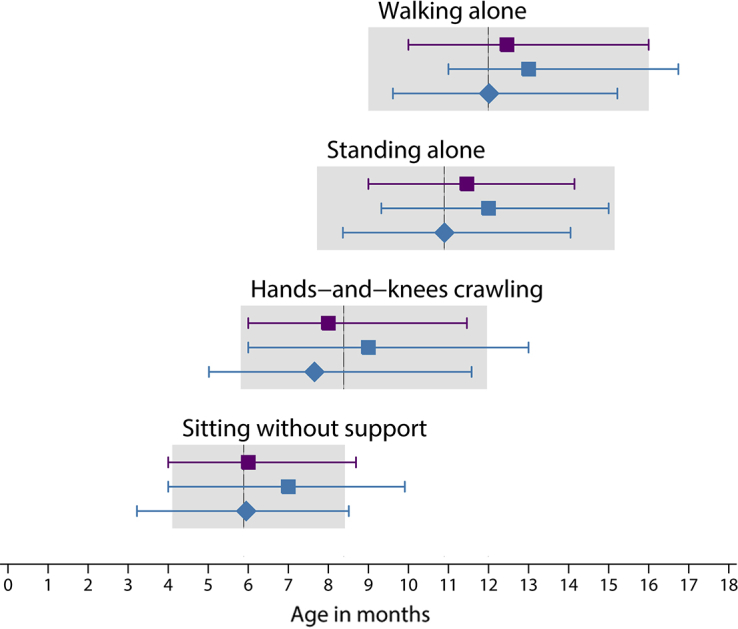
Median age of achievement (3rd and 97th percentiles) of 4 gross motor development milestones Data are for children who were included in the INTERGROWTH-21^st^ Fetal Growth Standards^21^ (*purple*) and children who were included in the INTERGROWTH-21^st^ Preterm Postnatal Growth Standards^22^ (*blue*). The *diamonds* represent the use of corrected age for the children who were born preterm. For comparison, the 3rd and 97th percentiles of the World Health Organization windows of achievement^35^ for the same milestones are presented in *grey* (with the median shown as a *vertical line*). *Villar et al. Validation of the INTERGROWTH-21^st^ fetal growth standards. Am J Obstet Gynecol 2018*.

The mean postnatal ages, at which the 4 main WHO milestones for gross motor development^29^ were achieved for the total cohort and preterm subgroup (chronologic and corrected age) are presented in Figure 3. Both groups overlapped well for these milestones at the 50th, 3rd, and 97th percentiles of the WHO range for normal term infants. By 2 years of age, >99% of the children had achieved the 4 motor development milestones with >97% within the range of the WHO milestones (data not shown). However, although the preterm subgroup overlapped very well when corrected age was used, they displayed a delay of approximately 1 month in achieving the “walking alone” and “standing alone” milestones, when estimated age after birth was used (Figure 3).

### The variability in children’s length among study sites compared with that among individuals within a study site

Maintaining the same analytic approach to the 2-year follow-up data that was adopted for the fetus and newborn infant,^4^, ^16^, ^37^ we summarized the variability in skeletal growth and size during pregnancy, at birth, and in infancy and childhood (ie, quantifying the variability among study sites, as opposed to that among individuals within a given site). We estimated that the variance among our study sites from birth through 1–2 years of age explains only 5.5% of the total variability in length between birth and 2 years of age; the variance among individuals within a study site explains 42.9% (ie, 8 times the amount after we controlled for age and sex). In Table 7, we compared the present results with the previously published INTERGROWTH-21^st^ data from the first trimester of pregnancy to 2 years of age. In all these periods of rapid growth, the variance among sites explains <10% of the total variability in skeletal growth.

**Table 7:** Variance components analysis for fetal, newborn infant, and childhood skeletal growth from the cohort of the INTERGROWTH-21^st^ Project

Variance	Fetal ultrasound measures^16^, %		Size at birth^16^ (newborn infant length^a^), %	Infancy/childhood, %	
	1st-trimester fetal crown-rump length^a^	2nd- and 3rd-trimester fetal head circumference		Preterm infant length^22^	Present study length^b^
Among study sites	1.9	2.6	3.5	0.2	5.5
Among individuals within a site	—	18.6	—	57.1	42.9
Residual	98.1	78.8	96.5	42.7	51.6

*Villar et al. Validation of the INTERGROWTH-21^st^ fetal growth standards. Am J Obstet Gynecol 2018*.

a Variance between individuals for these measures could not be estimated, given the cross-sectional nature of the data

b Includes length measurements at birth, 1 and 2 years, controlled for age and sex.

### Estimated adult height of the children included in the INTERGROWTH- 21^st^ fetal growth standards

**Figure 4:**
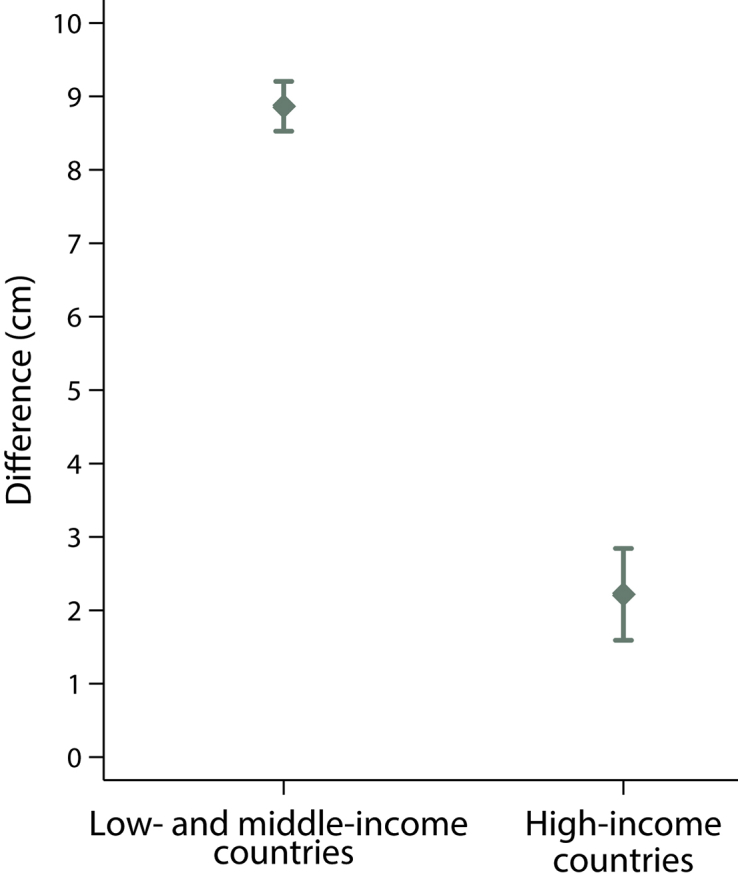
Expected increase from parental height Mean (95% confidence interval) difference between estimated adult height (calculated by doubling infant length at 2 years of age) and mean parental height (calculated as the average of maternal and paternal heights) for children who were included in the INTERGROWTH-21^st^ Fetal Growth Standards^21^ for study sites located in low- and middle-income countries and high-income countries. *Villar et al. Validation of the INTERGROWTH-21^st^ fetal growth standards. Am J Obstet Gynecol 2018*.

We estimated the difference between the observed mean parental height and the expected mean adult height (equal to approximately double the mean length at 2 years of age).^37^ In the study sites in low-middle income countries (n=1611), an increase in mean expected adult height of 8.9 cm over mean parental height is predicted to occur in a single generation, provided that infants and children are exposed to adequate health, environmental and nutritional conditions from early pregnancy onwards (Figure 4). Conversely, in high-income country sites (N=415), this cohort will be on average 2.2 cm taller than their parents (Figure 4).

## Comment

### Main findings

The participants included in the construction of the Fetal Growth Standards, the Newborn Size at Birth and the Preterm Postnatal Growth Standards of the INTERGROWTH-21^st^ Project were selected during early pregnancy specifically to generate international standards.^15^ The comprehensive data presented here, which describe for the first time the postnatal physical growth, infant mortality rate, morbidity and motor development of the INTERGROWTH-21^st^ participants, corroborate that they conformed to the WHO prescriptive approach for the construction of human growth standards. They are a cohort with continuous very low rates of clinical conditions that could affect optimal growth and development.

Our findings reinforce the a priori concept^17^ that it is possible to identify a subset of mostly moderate and late preterm infants, with no evidence of intrauterine growth restriction and limited neonatal morbidity,^41^ which constitutes an adequate approximation (in terms of growth, health, nutrition, and development) to a prescriptive population for the construction of preterm postnatal growth standards up to 64 weeks postmenstrual age, the time at which they match the WHO Child Growth Standards.^22^, ^23^

### Strengths and limitations of the study in the context of the existing literature

As far as we are aware, this is the first time that a fetal cohort that has been included in longitudinal studies for the specific purpose of constructing prescriptive growth standards has been evaluated up to 2 years of age. Most ultrasound studies that aimed to create reference charts for fetal growth have not reported any postnatal assessment, nor is it likely that such an assessment has been carried out, given the time that has elapsed since these studies were conducted.^9^ We selected the 2-year milestone because nutrition indicators that are measured at this age are strongly predictive of adult measures of nutrition, human capital, attained height, and intelligence.^42^ Before the age of 2 years, it has been shown that children often cross growth percentiles, whereas after this age the phenomenon known as “growth channelization” has been demonstrated, because children tend to grow along the same percentile.^20^

Our unique data are derived from a prospective follow-up evaluation of individuals from 7 different regions of the world from the first trimester of pregnancy to 2 years of age. These findings strengthen the case for the worldwide use of the international INTERGROWTH-21^st^ standards that complement the WHO Child Growth Standards in postnatal life. The similarities between the INTERGROWTH-21^st^ and WHO studies mean that the size of children, measured at 2 years of age, who were born to healthy mothers, with adequate nutrition, from healthy populations at low risk of adverse pregnancy outcomes, is consistent between the studies and across time.

Other strong features of the study include careful standardization of the outcome measures and the comprehensiveness of the standardized clinical and developmental assessments at 2 years of age.^43^ Furthermore, we followed 82% of the children and up to 86% of the preterm subgroup, which are excellent rates for free-living urban subjects. Baseline similarities between the infants who were evaluated and those lost to follow-up demonstrate that selection bias is very unlikely to have influenced the observed results.

We acknowledge some limitations that relate to practical difficulties of carrying out such a large, multicenter study.

First, the information on morbidity refers mostly to substantive clinical episodes, and data on gross motor development were obtained from parental report rather than direct observation; however, we informed parents about the Infant Follow-up Study protocol at enrolment and asked them to record severe conditions and infant developmental milestones. In addition, parents were encouraged to bring sick children for care to the participating centers; illness therefore was recorded at the time of the event.

Second, the study outcomes do not extend >2 years of age. This juncture was selected because it is a key time for the detection of postnatal growth faltering,^44^, ^45^ and an anthropometric and clinical evaluation at this age is a very good predictor of subsequent growth. Finally, the Seattle, WA, study site did not participate in the follow-up of children because of logistic issues that were associated with this inner city, highly mobile population. Although they represented only 298 newborn infants of more than 4000 in the total cohort and it is very unlikely that they would have affected the overall results presented here, it would have been better to have studied this subsample as well.

The WHO motor development assessment is a simple, pragmatic, and reliable tool to describe normal variation in the achievement of milestones that are reached progressively across infancy. It is especially recommended for studying a large number of infants at the cohort level, rather than individual level.^35^ We have observed that, using chronologic age, gross motor indicators for 2-year-old children who were born preterm (despite being always within WHO recommended windows) are below those of the total cohort by approximately 1 month. This pattern disappears with the use of corrected age (Figure 3). Thus, it is likely that the true range of development in uncomplicated preterm infants is between chronologic and corrected age. However, it is possible that levels of preterm postnatal development may be associated with etiologic phenotypes, as was shown with early neonatal morbidity.^41^ We presently are studying these issues in the INTERBIO-21^st^ Study, which is the extension to the INTERGROWTH-21^st^ Project.

In all the INTERGROWTH-21^st^ publications, we have emphasized that the relevant question when comparing growth across populations is whether the variability in skeletal growth within a population (interindividual genetic difference) is larger than the variability among populations (interpopulation genetic difference) when nutritional and health needs are met.

We have used variance components analysis in cohorts that were followed prospectively to identify the proportional contribution of the within and between sites variance components.^4^, ^16^, ^23^ We have repeated this analysis for the present article (Table 7). The variance from birth to 2 years of age within a geographic area is 8 times larger than that among geographic areas (Table 7). Hence, it is very unlikely that variability among geographic areas explains >10% of the total variability in infant and child length in healthy, well-nourished, low-risk populations who receive adequate healthcare. These results are in very close agreement with the data from the WHO Child Growth Standards for children <5 years of age, where the variability within study sites explained 70% of the total variance as opposed to a figure of 3.4% that is explained by the between-study sites variability.^23^

This clinical/epidemiologic finding is of great biologic interest because it is consistent with a metaanalysis of 22 genome-wide association studies that showed that the polygenic scores, based on 180 single nucleotide polymorphisms that previously were associated with adult height, explained only a very small proportion of the total variance in birth and infant length (0.13% and 2.95%, respectively).^46^

### Long-term implications

Our 2-year follow-up evaluation of this large cohort of healthy children allowed their mean predicted adult height to be estimated based on the assumption that health, nutritional, and socioeconomic conditions would remain adequate (Figure 4). Thus, the participants in the low-middle income countries sites (and by implication those from other similar countries) are expected to be approximately 8 cm taller as adults than the mean height of their parents; these data are very close to the 6.2–7.8 cm results that were observed in a similar, secondary analysis of the WHO Multicentre Growth Reference Study database.^37^ However, because optimal growth largely has been achieved in the parents from high-income country sites, their children are expected to be, on average, only 2.2 cm taller (Figure 4).

Our results confirm a pattern and magnitude of apparent transgenerational “washout”^47^ that has previously been described in the Multicentre Growth Reference Study populations.^37^ This effect on skeletal growth suggests that a highly sensitive response to environmental changes (eg, better intrauterine and infant nutrition and healthcare) can occur in 1 generation (ie, in a much shorter timeframe than evolution allows). The mechanisms, which may be mediated by modifications in gene expression that are not linked to DNA sequence changes, are being investigated currently at the molecular level in the INTERBIO-21^st^ Study.

The observation that this healthy cohort was at the 58th percentile of the sex-specific weight for age of the WHO Child Growth Standards at 2 years of age has potential implications in describing the natural history of becoming overweight among healthy infants. Because we did not implement any specific nutritional intervention, other than to promote breastfeeding, this weight distribution may represent the initial stages of the overweight epidemic facing many urban children who are exposed to westernized diets. Recent standardized, prospectively collected, fetal data have confirmed the complex effect of nutrition, the environment, migration, and social-cultural issues on fetal growth patterns.^48^, ^49^, ^50^

The short-term shift in weight distribution in an otherwise healthy population that we have described also reinforces the concept that comparisons among populations to evaluate growth potential should be based on length rather than weight because of its sensitivity to acute influences.

A larger question that goes beyond the scope of this article relates to the timing, velocity, and individual tracking of growth from conception to 2 years of age vis-à-vis feeding and morbidity in high-risk populations. The exploration of these questions in a longitudinal fashion, including interactions, has considerable statistical complexity, which we are presently investigating in the INTERBIO-21^st^ Study.

In summary, we have presented evidence that the participants who are enrolled in the international Fetal Growth Standards and the Preterm Postnatal Growth Standards of the INTERGROWTH-21^st^ Project and who were selected based on the WHO prescriptive approach for growth standards remain healthy and have adequate growth and development patterns at the key milestone of 2 years of age. This is additional strong confirmation of the sample’s appropriateness for the construction of international growth standards. The INTERGROWTH-21^st^ international standards are freely available (www.intergrowth21.tghn.org) for use worldwide.

### Contributors

J.V. and S.H.K. conceptualized and designed the INTERGROWTH-21^st^ Project. J.V., S.H.K., D.G.A., and A.J.N. prepared the original protocol, with later input from A.T.P., L.C.I., F.C.B., and ZAB. J.V., A.T.P., L.C.I., A.L., and Z.A.B. supervised and coordinated the project’s overall undertaking. E.S.U., E.O.O., and D.G.A. carried out data management and analysis in collaboration with J.V. R.P., F.C.B., R.O., Y.A.J., E.B., and M.P. collaborated in the overall project and implemented it in their respective countries. F.G. assisted in the global coordination of the project; L.C.I. and C.C. led the quality control of the anthropometric component, and M.F. and A.S. led the neurodevelopment assessment component. J.V. and S.K. wrote the report with significant contributions by F.G., C.G., C.G.V., F.C.B., and Z.A.B. All coauthors read the report and made suggestions on its content.

## Members of the International Fetal and Newborn Growth Consortium for the 21^st^ Century (INTERGROWTH-21^st^) and its Committees

Scientific Advisory Committee: M. Katz (Chair from January 2011), M.K. Bhan, C. Garza, S. Zaidi, A. Langer, P.M. Rothwell (from February 2011), Sir D. Weatherall (Chair until December 2010).

Steering Committee: Z.A. Bhutta (Chair), J. Villar (Principal Investigator), S. Kennedy (Project Director), D.G. Altman, F.C. Barros, E. Bertino, F. Burton, M. Carvalho, L. Cheikh Ismail, W.C. Chumlea, M.G. Gravett, Y.A. Jaffer, A. Lambert, P. Lumbiganon, J.A. Noble, R.Y. Pang, A.T. Papageorghiou, M. Purwar, J. Rivera, C. Victora.

Executive Committee: J. Villar (Chair), D.G. Altman, Z.A. Bhutta, L. Cheikh Ismail, S. Kennedy, A. Lambert, J.A. Noble, A.T. Papageorghiou.

Project Coordinating Unit: J. Villar (Head), S. Kennedy, L. Cheikh Ismail, A. Lambert, A.T. Papageorghiou, M. Shorten, L. Hoch (until May 2011), H.E. Knight (until August 2011), E.O. Ohuma (from September 2010), C. Cosgrove (from July 2011), I. Blakey (from March 2011).

Data Analysis Group: D.G. Altman (Head), E.O. Ohuma, E. Staines Urias (from April 2016), J. Villar.

Data Management Group: D.G. Altman (Head), F. Roseman, N Kunnawar, S.H. Gu, J.H. Wang, M.H. Wu, M. Domingues, P. Gilli, L. Juodvirsiene, L. Hoch (until May 2011), N. Musee (until June 2011), H. Al-Jabri (until October 2010), S. Waller (until June 2011), C. Cosgrove (from July 2011), D. Muninzwa (from October 2011), E.O. Ohuma (from September 2010), D. Yellappan (from November 2010), A. Carter (from July 2011), D. Reade (from June 2012), R. Miller (from June 2012), ESU (from April 2016).

Ultrasound Group: A.T. Papageorghiou (Head), L. Salomon (Senior external advisor), A. Leston, A. Mitidieri, F. Al-Aamri, W. Paulsene, J. Sande, W.K.S. Al-Zadjali, C. Batiuk, S. Bornemeier, M. Carvalho, M. Dighe, P. Gaglioti, N. Jacinta, S. Jaiswal, J.A. Noble, K. Oas, M. Oberto, E. Olearo, M.G. Owende, J. Shah, S. Sohoni, T. Todros, M. Venkataraman, S. Vinayak, L. Wang, D. Wilson, Q.Q. Wu, S. Zaidi, Y. Zhang, P. Chamberlain (until September 2012), D. Danelon (until July 2010), I. Sarris (until June 2010), J. Dhami (until July 2011), C. Ioannou (until February 2012), C.L. Knight (from October 2010), R. Napolitano (from July 2011), S. Wanyonyi (from May 2012), C. Pace (from January 2011), V. Mkrtychyan (from June 2012).

Anthropometry Group: L. Cheikh Ismail (Head), W.C. Chumlea (Senior external advisor), F. Al-Habsi, Z.A. Bhutta, A. Carter, M. Alija, J.M. Jimenez-Bustos, J. Kizidio, F. Puglia, N. Kunnawar, H. Liu, S. Lloyd, D. Mota, R. Ochieng, C. Rossi, M. Sanchez Luna, Y.J. Shen, H.E. Knight (until August 2011), D.A. Rocco (from June 2012), I.O. Frederick (from June 2012).

Neonatal Group: Z.A. Bhutta (Head), E. Albernaz, M. Batra, B.A. Bhat, E Bertino, P. Di Nicola, F. Giuliani, I. Rovelli, K. McCormick, R. Ochieng, R.Y. Pang, V. Paul, V. Rajan, A. Wilkinson, A. Varalda (from September 2012).

Environmental Health Group: B. Eskenazi (Head), L.A. Corra, H. Dolk, J. Golding, A. Matijasevich, T. de Wet, J.J. Zhang, A. Bradman, D. Finkton, O. Burnham, F. Farhi.

## Participating countries and local investigators

*Brazil:* F.C Barros (Principal Investigator), M. Domingues, S. Fonseca, A. Leston, A. Mitidieri, D. Mota, I.K. Sclowitz, M.F. da Silveira.

*China:* R.Y. Pang (Principal Investigator), Y.P. He, Y. Pan, Y.J. Shen, M.H. Wu, Q.Q. Wu, J.H. Wang, Y. Yuan, Y. Zhang.

*India:* M. Purwar (Principal Investigator), A. Choudhary, S. Choudhary, S. Deshmukh, D. Dongaonkar, M. Ketkar, V. Khedikar, N. Kunnawar, C. Mahorkar, I. Mulik, K. Saboo, C. Shembekar, A. Singh, V. Taori, K. Tayade, A. Somani.

*Italy:* E. Bertino (Principal Investigator), P. Di Nicola, M. Frigerio, G. Gilli, P. Gilli, M. Giolito, F. Giuliani, M. Oberto, L. Occhi, C. Rossi, I. Rovelli, F. Signorile, T. Todros.

*Kenya:* W. Stones and M. Carvalho (Co- Principal Investigators), J. Kizidio, R. Ochieng, J. Shah, S. Vinayak, N. Musee (until June 2011), C. Kisiang’ani (until July 2011), D. Muninzwa (from August 2011).

*Oman:* Y.A. Jaffer (Principal Investigator), J. Al-Abri, J. Al-Abduwani, F.M. Al-Habsi, H. Al-Lawatiya, B. Al-Rashidiya, W.K.S. Al-Zadjali, F.R. Juangco, M. Venkataraman, H. Al-Jabri (until October 2010), D. Yellappan (from November 2010).

*UK:* S. Kennedy (Principal Investigator), L. Cheikh Ismail, A.T. Papageorghiou, F. Roseman, A. Lambert, E.O. Ohuma, S. Lloyd, R. Napolitano (from July 2011), C. Ioannou (until February 2012), I. Sarris (until June 2010).

*USA:* M.G. Gravett (Principal Investigator), C. Batiuk, M. Batra, S. Bornemeier, M. Dighe, K. Oas, W. Paulsene, D. Wilson, I.O. Frederick, H.F. Andersen, S.E. Abbott, A.A. Carter, H. Algren, D.A. Rocco, T.K. Sorensen, D. Enquobahrie, S. Waller (until June 2011).

## Figures and Tables

**Supplementary Figure:**
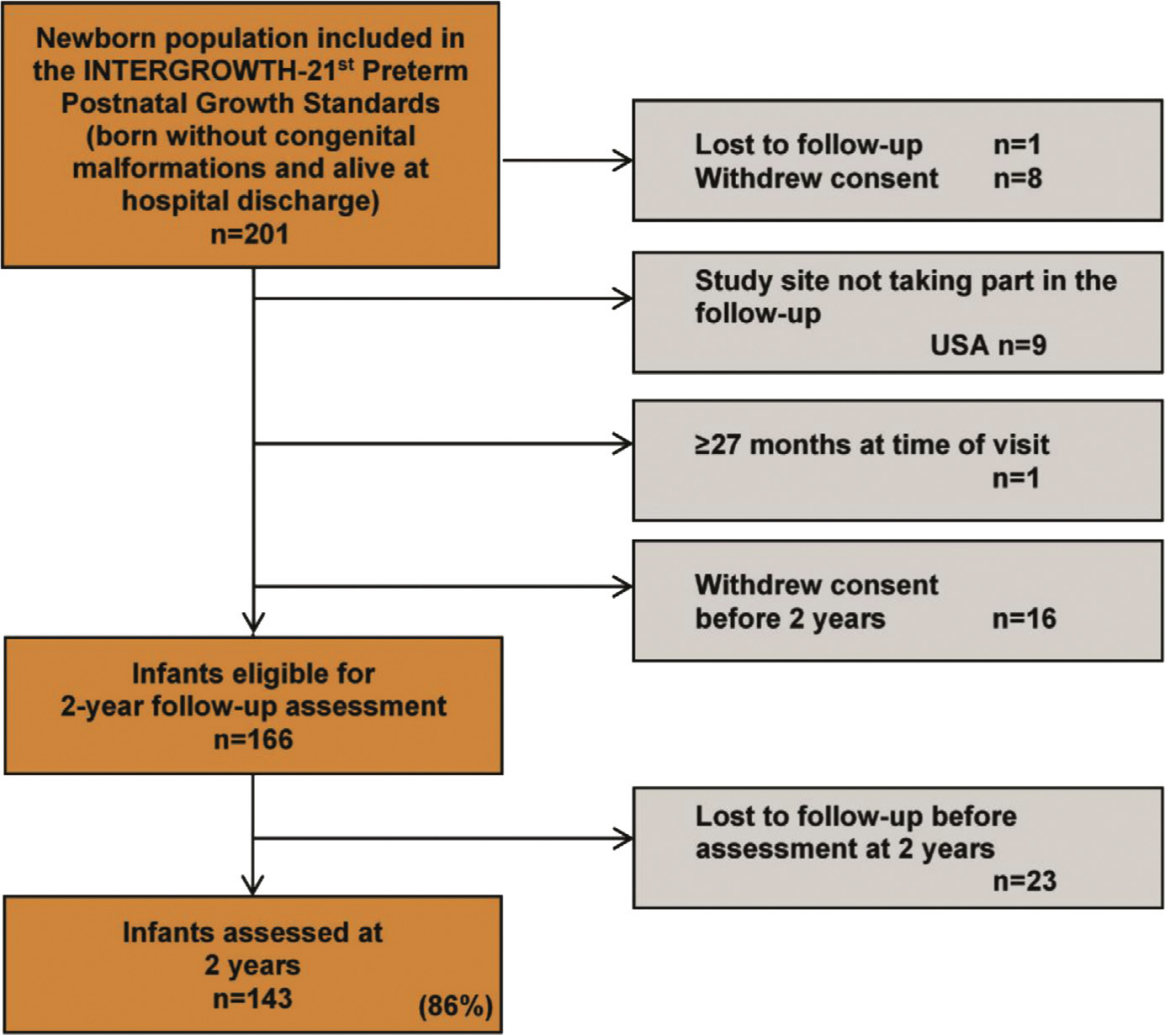
Study flow of INTERGROWTH-21^st^ Preterm Postnatal Follow-up at 2 years The chart shows the cohort that contributed data to the construction of the INTERGROWTH-21^st^ Preterm Postnatal Growth Standards.^22^ *Villar et al. Validation of the INTERGROWTH-21^st^ fetal growth standards. Am J Obstet Gynecol 2018*.

**Supplementary Table 1:** Twenty-four–hour dietary intake of children who were included in the INTERGROWTH-21^st^ Fetal Growth Standards^21^ at 1 and 2 years of age

Food group given to the child at least once a day	1 Year of age (n=2832), n (%)	2 Years of age (n=3041), n (%)
Grains, roots, and tubers	2811 (99.3)	3031 (99.7)
Legumes and nuts	1124 (39.7)	1375 (45.2)
Dairy products	2822 (99.6)	3040 (100.0)
Flesh foods	1676 (59.2)	2083 (68.5)
Eggs	575 (20.3)	889 (29.2)
Vitamin-A-rich fruits	1907 (67.3)	1950 (64.1)
Other fruits and vegetables	2606 (92.0)	2863 (94.1)
Fats: spreads/oils	885 (31.3)	1342 (44.1)
Sugars: sweets/sugar products/jelly/sweetened drinks	435 (15.4)	989 (32.5)

*Villar et al. Validation of the INTERGROWTH-21^st^ fetal growth standards. Am J Obstet Gynecol 2018*.

**Supplementary Table 2:** Twenty-four–hour dietary intake for children who were included in the INTERGROWTH-21^st^ Preterm Postnatal Growth Standards^22^ evaluated at 1 and 2 years of age

Food group given to the child at least once a day	1 Year of age (n=154)	2 Years of age (n=143)
Grains, roots, and tubers	153 (99.4)	142 (99.3)
Legumes and nuts	54 (35.1)	68 (47.6)
Dairy products	154 (100.0)	143 (100.0)
Flesh foods	95 (61.7)	101 (70.6)
Eggs	35 (22.7)	35 (24.5)
Vitamin-A-rich fruits	104 (67.5)	84 (58.7)
Other fruits and vegetables	130 (84.4)	133 (93.0)
Fats: spreads/oils	34 (22.1)	55 (38.5)
Sugars: sweets/sugar products/jelly/sweetened drinks	14 (9.1)	45 (31.5)

*Villar et al. Validation of the INTERGROWTH-21^st^ fetal growth standards. Am J Obstet Gynecol 2018*.
